# Hepatic Hilar Block as an Adjunct to Transarterial Embolization of Neuroendocrine Tumors: A Retrospective Review of Safety and Efficacy

**DOI:** 10.3390/cancers15215202

**Published:** 2023-10-29

**Authors:** Samagra Jain, Harrison Blume, Lee Rodriguez, Elena Petre, Amgad Moussa, Ken Zhao, Vlasios Sotirchos, Nitya Raj, Diane Reidy, Etay Ziv, Erica Alexander

**Affiliations:** 1 Department of Radiology, Memorial Sloan Kettering Cancer Center, 1275 York Ave, New York, NY 10065, USA; jains4@mskcc.org (S.J.); blumeh@mskcc.org (H.B.); rodrl10@mskcc.org (L.R.); petree@mskcc.org (E.P.); moussaa@mskcc.org (A.M.); zhaok@mskcc.org (K.Z.); sotirchv@mskcc.org (V.S.); zive@mskcc.org (E.Z.); 2 Baylor College of Medicine, School of Medicine, One Baylor Plaza, Houston, TX 77004, USA; 3 Albert Einstein College of Medicine, School of Medicine, 1300 Morris Park Ave, Bronx, NY 10461, USA; 4 Department of Medicine, Memorial Sloan Kettering Cancer Center, 1275 York Ave, New York, NY 10065, USA; rajn@mkscc.org (N.R.); reidyd@mskcc.org (D.R.)

**Keywords:** interventional oncology, neuroendocrine tumors, post-embolization syndrome, retrospective review

## Abstract

**Simple Summary:**

Locoregional transarterial therapies such as transarterial embolization are essential in the treatment of neuroendocrine tumors. However, side effects of embolization, such as nausea, pain, and fever (collectively termed post-embolization syndrome), are significant and can adversely affect patient recovery and post-procedural quality of life. This study aimed to determine if administration of a periprocedural hepatic hilar nerve block as an analgesic would be significant in affecting post-procedural pain and quality of life metrics. Management of post-embolization syndrome is necessary for optimal patient comfort and recovery, and this research fills a critical gap in this area of study.

**Abstract:**

Purpose: This study investigates whether hepatic hilar nerve blocks (HHNB) provide safe, effective analgesia in patients with neuroendocrine tumors (NET) treated with transarterial embolization (TAE). Methods: The retrospective study included all NETs treated with TAE or TAE + HHNB from 1/2020 to 8/2022. Eighty-five patients (45 men), mean age 62 years, were treated in 165 sessions (TAE, *n* = 153; TAE + HHNB, *n* = 12). For HHNBs, ≤10 mL bupivacaine HCl 0.25% ± 2 mg methylprednisolone were injected under ultrasound guidance. The aims were to assess safety of HHNB and reduction in pain. Groups were compared with Pearson’s chi-squared and Wilcoxon rank sum tests. Logistic regression assessed independent risk factors for pain. Results: No immediate complications from HHNBs were reported. No difference in incidence of major complications between TAE and TAE + HHNB one month post-embolization was observed (7.19% vs. 8.33%, *p* = 0.895). No differences in mean length of hospital stay after treatment were observed (TAE 2.2 days [95%CI: 1.74–2.56] vs. TAE + HHNB 2.8 days [95%CI: 1.43–4.26]; *p* = 0.174). Post-procedure pain was reported in 88.2% of TAE and 75.0% of TAE + HHNB patients (*p* = 0.185). HHNB recipients were more likely to use analgesic patches (25.0% vs. 5.88%; *p* = 0.014). No other differences in analgesic use were observed. Conclusions: HHNBs can safely be performed in patients with NETs. No difference in hospital stays or analgesic drug use was observed. Managing pain after TAE is an important goal; further study is warranted.

## 1. Introduction

Neuroendocrine tumors (NETs) represent a heterogeneous family of neoplasms, arising primarily from the gastrointestinal tract, pancreas, and lung. Approximately 175,000 people in the United States are living with NETs [1]. Rates of diagnosis continue to increase each year, likely due to improvements in diagnosis and awareness. At the time of diagnosis, many patients present with lymph node and liver metastases, which prognosticate worse survival outcomes [2,3]. Over the past three decades, locoregional transarterial therapies for NET liver metastases, such as transarterial embolization (TAE), have been widely adopted to achieve tumor and symptom control. Studies of NET embolization have shown effective control of carcinoid symptoms, improvement in progression-free survival, and improvement in overall survival [4,5,6,7,8]. TAE has also been demonstrated to be safe and well-tolerated for the treatment of NETs, resulting in this treatment becoming a prominent primary therapeutic option for this disease. However, embolization is not without side effects, with up to 90% of patients reporting post-embolization syndrome (PES), a compilation of symptoms including nausea, vomiting, anorexia, abdominal pain, fever, and leukocytosis after treatment [9]. These symptoms typically self-resolve within 24 h of the procedure in standard cases but may persist for up to two weeks, according to some reports [10]. The underlying pathophysiology of PES has yet to be completely elucidated, but the primary mechanism is believed to be linked to tissue and tumor ischemia, resulting in cell death followed by cytokine release and local/systemic immune response [11].

A major obstacle in managing PES in patients with NETs is that there is no standardization in the literature or clinical practice. Current practice is heavily reliant on the use of opioid medications for post-procedural pain management, which are associated with numerous complications and side effects, including cardiopulmonary depression, addiction/reliance, nausea/vomiting, altered mental status and gastrointestinal complications [12]. Although post-embolization syndrome is generally considered a minor and expected side effect of TAE, symptoms can be debilitating and adversely affect patient health and well-being in the short and long term. In addition to increasing patient opioid dependence, PES symptoms can also prolong hospital admission and decrease patient comfort and satisfaction, resulting in further costs and burdens on healthcare systems [13]. Along with providing patients with symptomatic relief, it is critical to explore interventions that can decrease and even prevent PES from occurring after embolization procedures. Several such non-opioid therapies and techniques have been described to help mitigate PES, including intravenous N-acetylcysteine, intravenous dexamethasone, intraarterial dexamethasone, intraarterial lidocaine, and specific peripheral nerve blocks such as celiac, paravertebral, and hepatic hilar nerve blocks (HHNBs) [14,15,16,17,18,19,20]. Peripheral nerve blocks are an appealing adjunct to embolization therapies as they represent a non-opioid analgesic option, which is quick, inexpensive, and effective. Receipt of periprocedural peripheral nerve blocks has been associated with a reduced length of hospitalization, lower maximum pain ratings, and decreased opioid use in a variety of orthopedic procedures [21]. However, little data exist about the specific use of HHNBs for interventional oncology procedures such as TAE [22,23]. The aim of this study is to establish the safety and efficacy of hepatic nerve blocks as an adjunct to the embolization of NETs.

## 2. Materials and Methods

This retrospective study was approved by the Institutional Review Board and is compliant with the Health Insurance Portability and Accountability Act. Written informed consent was waived.

### 2.1. Patient Demographics and Data Collection

This study included all NET patients treated with TAE alone or TAE + HHNB at a single specialized tertiary care institution between January 2020 and August 2022. A total of 85 patients (45 men), with a mean age of 62 years (range 36–87) were treated in 165 sessions (TAE, *n* = 153; TAE + HHNB, *n* = 12). A variety of preprocedural and postprocedural variables were collected and assessed from the electronic medical record and picture archiving and communication system. Preprocedural variables included age, prior liver interventions, presence of extrahepatic disease, liver treatment data (size of lesions, bilobar disease, number of lesions, and percent of liver involvement) and use of pre-existing pain medication(s) (opioid vs. non-opioid). Postoperative variables included length of hospital stay, development of nausea and use of antiemetics, development of pain and use of pain relievers and/or opioids, and unplanned rehospitalizations within thirty days. Of note, the length of hospital stay was determined for those patients who were admitted to the hospital at the time of the procedure. Nine patients treated over 12 sessions were admitted prior to treatment for symptoms secondary to neuroendocrine tumors, including hypoglycemia, heart failure, dehydration, and liver dysfunction. These encounters were not included in calculations of the overall length of stay. Complications were graded according to the SIR Adverse Events Classification [24].

### 2.2. Treatment Technique

Patients were referred to the interventional radiology (IR) department by medical oncologists in the gastrointestinal oncology division and were evaluated in IR clinic prior to treatment. All patients were required to have updated cross-sectional imaging, including computed tomographic (CT) imaging and/or magnetic resonance imaging, and updated laboratory values, specifically complete metabolic panel, complete blood count, and coagulation factors. Inclusion criteria included a platelet count ≥ 50, an international normalized ratio ≤ 1.5, and total bilirubin levels ≤ 2. One hour prior to treatment, all patients received 250 mcg of subcutaneous octreotide and 1 g of IV cefazolin for prophylaxis; those patients with a documented penicillin allergy received clindamycin 900 mg IV and gentamicin 1.5 mg/kg IV. All procedures, including HHNBs, were performed under moderate sedation, monitored anesthesia care, or general anesthesia.

**Figure 1:**
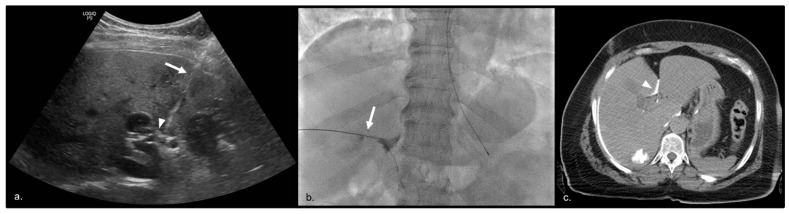
Using ultrasound guidance, a 22-gauge needle (arrow) was placed at the bifurcation of the main portal vein (arrowhead) (**a**). Contrast was injected into the 22-gauge needle (arrow) under fluoroscopy to confirm extravascular location (**b**). CT imaging was then obtained, which showed contrast surrounding the portal vein (arrowhead) (**c**).

Prior to the embolization, those patients undergoing a HHNB were positioned supine and had a 21- or 22-gauge spinal needle directed towards the periportal fat at the portal bifurcation with ultrasound guidance. Aspiration was used to confirm the absence of intravascular communication, and contrast was injected under fluoroscopic guidance to confirm extravascular location. A solution containing up to 10 mL of 0.25% bupivacaine with or without 2 mg of methylprednisolone was injected into the hepatic nerve plexus and the needle was subsequently removed. Appropriate localization of the solution in the hepatic hilum was confirmed under CT imaging (Figure 1).

Transarterial embolization procedures were performed by a board-certified interventional radiologist, as previously described in the literature [25]. Embolization therapies were performed with polymer-based Embospheres (40–120 μm, 100–300 μm, 300–500 μm, 500–700 μm, Merit Medical, South Jordan, Utah) and/or Polyvinyl alcohol (PVA) foam (100 μm, 300 μm, Cook Medical, Bloomington, Indiana). Embolic choice was based on the preference of the provider, with consideration for tumor volume and the presence of portal venous shunting. Embolization was performed to stasis of the intended vessel(s), whilst avoiding reflux into non-target vasculature.

Post-procedure, patients were admitted for pain control and monitoring. Patients were treated with oral and/or intravenous pain medications, including, ketorolac, acetaminophen, opioids, analgesic patches, and/or hydromorphone patient-controlled anesthesia (PCA) pump, as needed and as determined by the treating provider. Antiemetics were administered if patients reported nausea or had episodes of emesis. Discharge timing was determined based on patients’ ability to tolerate a diet and control pain with oral analgesics. For those patients admitted with severe symptoms secondary to hormone secretion of their NETs, discharge was based on resolution of their presenting symptom(s).

### 2.3. Statistical Analysis

Outcomes assessed to evaluate the safety and efficacy of HHNB included rates of adverse events and complications associated with HHNB use, the association between HHNB use and post-procedural quality of life (QoL) measures, including pain reduction and hospital stay length, and the effect of HHNBs on post-procedural analgesic use.

Groups were compared using Pearson’s chi-squared and Wilcoxon rank sum tests. Univariate logistic regression was used to assess a variety of independent risk factors (extent of disease, embolization sphere size, and addition of a nerve block) for pain. For each regression, an odds ratio, 95% confidence interval, and *p*-value were obtained. Variables with a *p*-value less than or equal to 0.05 were considered statistically significant. Statistical analysis was accomplished with the use of Stata, version17 (StataCorp, College Station, TX, USA).

## 3. Results

### 3.1. Pre-Procedural Findings

Demographic information and pre-procedural variables, including prior liver interventions, extrahepatic metastases, and pre-procedural pain medications are summarized in Table 1. Patients in both groups were similar, except for the percentage of liver replaced with tumor. A greater percentage of patients in the embolization-only group had <50% of liver replaced with tumor (83.66%), whereas in the HHNB group, there was a more similar distribution of patients in the <50% vs. ≥50% tumor volume (58.33% vs. 41.67%, respectively). Of note, only one patient received a steroid injection in their nerve block, with all remaining patients receiving bupivacaine alone.

### 3.2. Post-Procedural Findings

Post-procedural QoL metrics related to post-embolization syndrome, including the presence of nausea (along with the extent of use of antiemetic medications), subjective reports of pain, and post-procedural use of oral and intravenous pain medications (NSAIDs, acetaminophen, opioids, analgesic patches and PCA) are presented in Table 2. Most procedures resulted in pain and nausea, and HHNBs did not have a significant effect on these variables. There were no significant differences in pain medication use between groups, except for analgesic patches, where 25% of the HHNB group reported utilization versus 5.9% of the embolization-only group (*p* = 0.014).

Data regarding post-procedural hospital length (accounting for pre-existing hospitalization), rehospitalization within 30 days, and complication data are shown in Table 3. The rehospitalization data differentiate between planned and unplanned admissions, as several patients presented for retreatment of the contralateral lobe in less than 30 days. No significant differences were seen between groups. Most procedures resulted in SIR Grade A complications, generally referring to mild post-embolization syndrome that was self-limited.

Logistic regressions for independent factors for post-procedural pain are presented in Table 4 with the corresponding odds ratios and 95% confidence intervals. The extent of disease, embolization sphere size, and addition of a nerve block were not found to have a significant impact on post-procedural pain reduction.

## 4. Discussion

This study aimed to determine if HHNBs provided safe, effective analgesia in patients with neuroendocrine tumors treated with TAE. Logistic regression demonstrated no significant association between HHNB treatment and post-procedure pain. Additionally, the use of HHNBs did not correlate with shorter hospital length of stay or reduced rehospitalization rate in this small, retrospective study. There were no immediate complications of HHNBs reported, and after one-month post-embolization, there were no significant differences in the incidence of major adverse events as classified by the Society of Interventional Radiology Adverse Events Criteria.

There was no significant decrease in post-procedure opioid analgesic use, but analgesic patches were more frequently utilized by patients after an HHNB (25% vs. 5.88%, *p* = 0.014). Of note, there was a larger percentage of patients in the HHNB group with tumor volume ≥ 50% of the liver volume, which may have resulted in worse post-procedure pain due to the larger volume of tumor treated and larger subsequent tissue ischemia. This may have resulted in the similar use of post procedure analgesics between both study groups, despite the additional analgesic effect offered by the nerve block, albeit this is largely speculative.

A 2021 study by Bessar et al. of 92 patients receiving HHNBs alongside doxorubicin-eluting embolic transarterial chemoembolization (DEB-TACE) therapy for hepatocellular carcinoma (HCC) also found no significant differences in post-procedure hospital stay length or adverse event incidence, but groups did observe improvements in pain ratings and opioid consumption [19]. An earlier study, published by Coldwell et al., evaluated the effect of celiac plexus blocks on intra- and post-procedure pain during and after TAE. The authors found that for those patients who received celiac plexus blocks, no narcotics were used during the procedure or for the first 8 h afterward. In the 8 to 24 h post-procedure, those patients who underwent the block had significantly less use of intravenous morphine compared to those patients without the nerve block [22]. Another study of thermal ablation of hepatic tumors found that HHNBs significantly decreased the postoperative use of fentanyl and midazolam without impacting procedural success or adverse event rates [26]. These similarities in clinical outcomes in both referenced studies suggest that hepatic plexus nerve blocks can decrease utilization of postoperative opioid utilization in patients undergoing liver directed therapy. Although the current study rendered largely negative results, likely secondary to the small treatment group size, it does suggest that HHNBs can be safely administered to patients undergoing TAE for NET treatment and that post-procedural QoL measurements are multifactorial, warranting further study on the viability of HHNBs as a periprocedural analgesic over the established standard of care.

The evaluation of QoL following interventional radiology/interventional oncology procedures is an important undertaking that results in a greater understanding of pain palliation and allows for a longitudinal focus on patients’ post-procedure pain and well-being. Measurement and analysis of health related QoL measures is an integral element of disease management. Unfortunately, very little research and attention has been focused on quality-of-life measures after liver directed therapies. Salem and colleagues conducted a multicenter observational cohort pilot study in patients with HCC who underwent transarterial radioembolization and collected serial patient-reported outcomes to assess health-related QoL [27]. The study was important for several reasons; firstly, it demonstrated the feasibility of collecting QoL outcomes from patients using a digital platform, and secondly, it highlighted that QoL measures are underutilized in assessing liver-directed procedures. While PES is typically a self-limited process, it can result in prolonged hospitalizations, repeat hospitalizations, and short-term impairment in functionality after procedures, all of which decrease QoL post-procedure. Better assessment of QoL via patient surveys and prospective pain scores is essential to adequately capture this data. Additionally, these procedures undoubtedly create expenditures on the healthcare system secondary to inpatient stays and readmissions and result in costs to patients due to delays in a return to daily life. PES remains poorly understood and understudied, despite its near ubiquitous occurrence after hepatic artery embolization. Further study of the inflammatory markers produced during embolization and the potential mediation of inflammation with nerve blocks would be beneficial to understanding PES on a molecular level. Additionally, greater focus on treatment strategies to shorten the duration and severity of PES is greatly needed.

Several research studies have been performed in the past examining the efficacy, safety, and long-term outcomes of TAE; while these studies have been critical to establishing TAE as an effective treatment modality for neuroendocrine tumors, there has been limited literature on the definitive mitigation and management of PES even though it is the most frequently reported complication of TAE [28]. In addition to improving patient QoL and healthcare costs, reduction of PES may have implications on overall survival in addition to patient QoL. In a retrospective study of 144 patients with HCC treated with TAE, multivariate analysis associated PES symptoms with a statistically significant two-fold increased risk of death (*p* = 0.011) [29]. It is important to note that the presence of bilobar compared to unilobar disease and use of DEB-TACE compared to conventional TACE were identified as independent predictors of PES in the aforementioned study. The former variable, namely bilobar disease, is often a proxy for disease volume and extent and may portend a worse prognosis in isolation. Additionally, a 2011 study from Romero Ubillus et al. showed a general correlation between PES and worse progression-free survival, albeit this finding was not significant [30]. However, it is important to note that patients with higher disease burden are more likely to experience PES and inherently have worse prognoses due to the extent of disease; as such, these conclusions should be viewed with caution.

The safety results of HHNBs in this study, particularly the lack of serious immediate adverse events or increase in hospitalization time/readmission rates, remain promising for the implementation of HHNB as an adjunct analgesic for TAE. HHNBs have reported advantages over celiac and paravertebral nerve blocks, including greater liver specificity and separation from critical structures such as the lungs and spine [31]. Additionally, compared to celiac plexus blocks, HHNBs are less likely to reach the bowel and can be performed using ultrasound and a supine patient position. The ease of use and supine positioning make HHNBs more likely to be utilized in the same setting as an embolization. However, prior studies have demonstrated that HHNBs have unique limitations as well. He et al. identified patient variant hepatic anatomy, such as the right phrenic artery bypassing the hepatic hilum into the liver, as a potential drawback in the efficacy of the standard HHNB procedure [32]. When utilizing this procedure, it is important to consider and adjust for unique anatomy to avoid unintended consequences. When using bupivacaine, a highly potent local nerve block anesthetic, it is crucial to ensure that it does not inadvertently accumulate outside of the intended treatment area and migrate to the cardiopulmonary or nervous systems, a complication which may result in severe ventricular arrhythmia, myocardial toxicity, or loss of nerve conduction [33]. Adequate operator training is critical to ensure the safety and success of this procedure.

The design of this study had multiple limitations. Perhaps the greatest limitation to this study was the sample size discrepancy between the HHNB + embolization group (*n* = 12) and the non-HHNB embolization group (*n* = 153). The small number of patients who received nerve blocks likely provided insufficient power to properly assess the impact of HHNBs on post-procedure pain and QoL measures. While other studies in the literature have shown added benefits to incorporating nerve blocks to liver-directed therapies, the lack of significant findings in the current study may be attributable to the study being underpowered. The retrospective nature and the variation in the treatment of post-procedure pain across patients, namely the lack of standardization in analgesics offered and/or dose escalation, is an inherent flaw. Additionally, there were variations in the medications delivered in the HHNB, with one patient receiving steroids. Of note, utilization of steroids in nerve blocks allows for longer-term mitigation of inflammation and pain, as bupivacaine has a short half-life of 1.5 to 5.5 h. Validation of the utility of nerve blocks in combination with embolization would require a prospective study, where patients receive a standardized post-procedure pain regimen. While there were no significant differences in post-embolization opioid use in this study, the nearly five-fold increase in HHNB patients using analgesic patches remains an unexplained result, but the small sample size of the HHNB group could be one contributing factor. Future studies with larger study groups and performed in a prospective nature would provide a more robust analysis.

## 5. Conclusions

HHNBs are a promising periprocedural intervention for mitigating PES following NET embolization. While this small, retrospective study did not identify an association between HHNB use and decreased post-procedural pain, hospital length of stay, readmission rates, or opioid use relative to the standard of care, this study did highlight that HHNBs are a safe and well-tolerated modality of pain control for NET embolization. Measurement of patient QoL is a complex yet important aspect of interventional radiology procedures, and future studies should continue to identify strategies to mitigate patient discomfort.

## Figures and Tables

**Table 1:** Demographic information.

	Overall (*n* = 165)	Embo + Nerve Block (*n* = 12)	Embo (*n* = 153)	*p*-Value
Age (mean, 95% CI)	62 (59–64)	62 (51–74)	62 (59–64)	0.934
Prior liver interventions (ex. Y90, ablation, embolization)				0.315
Yes	87 (52.73%)	8 (66.67%)	79 (51.63%)	
No	78 (47.27%)	4 (33.33%)	74 (48.37%)	
Liver treatment data				
Size of largest lesion in treated region (mean cm, SD)	5.20 (3.37)	5.15 (0.27)	5.88 (1.18)	0.472
Bilobar disease present				0.834
Yes	155 (93.88%)	12 (100%)	143 (93.46%)	
No	10 (6.12%)	0 (0%)	10 (6.54%)	
Number of hepatic lesions				0.810
1	2 (1.21%)	0 (0%)	2 (1.31%)	
2–5	27 (16.36%)	3 (25%)	24 (15.69%)	
>5	136 (82.43%)	9 (75%)	127 (83.0%)	
Percent of liver involved				0.020
>50%	28 (16.97%)	5 (41.67%)	23 (15.03%)	
<50%	137 (83.03)	7 (58.33%)	130 (74.97%)	
Extrahepatic liver metastases				
Yes	133 (80.61%)	11 (91.67%)	122 (79.74%)	0.314
No	32 (19.39%)	1 (8.33%)	31 (20.26%)	
Pre-procedure pain medication				
Yes	82	8 (66.67%)	74 (48.37%)	0.222
No	83	4 (33.33%)	79 (51.63%)	
Opioids	55	4 (50%)	51 (68.92%)	0.279
Non-opioids	27	4 (50%)	23 (31.08%)	

**Table 2:** Post-procedural quality of life and pain control metrics.

	Overall (*n* = 165)	Embo + Nerve Block (*n* = 12)	Embo (*n* = 153)	*p*-Value
Post-procedure nausea				0.347
Yes	128	8 (66.67%)	120 (78.43%)	
No	37	4 (33.33%)	33 (21.57%)	
Post-procedure antiemetic use				0.258
Yes	131	8 (66.67%)	123 (80.39%)	
No	34	4 (33.33%)	30 (19.61%)	
				0.856
1 antiemetic drug	106	8 (66.67%)	98 (64.05%)	
>1 drug	59	4 (33.33%)	55 (35.95%)	
Post-procedure pain				0.185
Yes	144	9 (75.0%)	135 (88.24%)	
No	21	3 (25.0%)	18 (11.76%)	
Number of analgesic drugs (mean, 95%CI)	2.85 (2.55–3.16)	2.83 (1.23–4.43)	2.86 (2.54–3.17)	0.8783
Post-procedure oral NSAID				0.293
Yes	13	0 (0%)	13 (8.5%)	
No	152	12 (100%)	140 (91.5%)	
Post-procedure IV NSAID				0.594
Yes	67	4 (33.33%)	63 (41.8%)	
No	98	8 (66.67%)	90 (58.82%)	
Number of IV NSAID doses (mean, 95% CI)	3.04	2.5 (1.29–6.29)	3.08 (2.34–3.82)	0.319 *
Post-procedure oral acetaminophen				0.462
Yes	53 (32.12%)	5 (41.67%)	48 (31.37%)	
No	112 (67.88%)	7 (58.83%)	105 (68.63%)	
Number of oral acetaminophen doses (mean, 95% CI)	2.98 (2.19–3.78)	1.8 (0.18–3.42)	3.10 (2.23–3.97)	0.274 *
Post-procedure IV acetaminophen				0.927
Yes	57 (34.55%)	4 (33.33%)	53 (34.64%)	
No	108 (65.45%)	8 (66.67%)	100 (65.36%)	
Number of IV acetaminophen doses (mean, 95% CI)	1.68	1 (1–1)	1.74 (1.16–2.31)	0.485 *
Post-procedure opioids oral				0.263
Yes	107 (64.85%)	6 (50%)	101 (66.01%)	
No	58 (35.15%)	6 (50%)	52 (33.99%)	
Number of opiates oral doses (mean, 95% CI)	4.47 (3.76–5.13)	5.67 (1.87–9.46)	4.40 (3.67–5.13)	0.230 *
Post-procedure opiates IV				0.455
Yes	93 (56.36%)	8 (66.67%)	85 (55.56%)	
No	72 (43.64%)	4 (33.33%)	68 (44.44%)	
Number of opiates IV doses (mean, 95% CI)	4.34 (3.32–5.37)	4.25 (0.60–7.90)	4.35 (3.26–5.45)	1.00 *
Post-procedure PCA				0.506
Yes	18 (10.91%)	2 (16.67%)	16 (10.46%)	
No	147 (89.09%)	10 (83.33%)	137 (89.54%)	
Number of PCA doses (mean, 95% CI)	7.44	4 (21.41–29.41)	7.88 (1.41–14.33)	0.723 *
Analgesic patch				0.014
Yes	12 (7.27%)	3 (25.0%)	9 (5.88%)	
No	153 (92.73%)	9 (75.0%)	144 (94.12%)	

* = Rank-sum test.

**Table 3:** Hospitalization and procedural tolerance data.

	Overall (*n* = 165)	Embo + Nerve Block (*n* = 12)	Embo (*n* = 153)	*p*-Value
Length of hospital stay (days, mean, 95% CI)	2.2 (0.81–2.17)	2.8 (1.43–4.26)	2.2 (1.74–2.56)	0.174 *
Length of hospital stay—pre-existing medical issues excluded (days, mean, 95% CI)	1.73 (1.52–1.93)	1.75 (0.59–2.91)	1.72 (1.51–1.94)	0.947 *
Rehospitalization within 30 days				0.839
For planned repeat	8 (4.85%)	1 (8.33%)	7 (4.58%)	
For complications	13 (7.88%)	1 (8.33%)	12 (7.84%)	
No hospitalization	144 (87.27%)	10 (83.33%)	134 (87.58%)	
SIR-grade complications				0.895
A	128 (77.85%)	9 (75%)	119 (77.78%)	
B	9 (5.45%)	1 (8.33%)	8 (5.23%)	
C	5 (3.03%)	0	5 (3.27%)	
D	6 (3.64%)	1 (8.33%)	5 (3.27%)	
F	1 (0.61%)	0	1 (0.65%)	
None	16 (9.7%)	1 (8.33%)	15 (9.8%)	

* = Rank-sum test.

**Table 4:** Independent logistic regressions for post-procedural pain.

Factor	OR	95% CI:	*p*-Value
Presence of extrahepatic metastases	1.813	0.643–5.124	0.260
Size of largest lesion in treated region	1.147	0.962–1.365	0.125
Bilobar disease	0.751	0.090–6.240	0.790
Liver involvement ≥50%	5.044	0.649–39.147	0.122
Treated region (≥ 4 segments)	1.672	0.669–4.209	0.269
Treatment of phrenic artery	1.491	0.181–12.294	0.710
Number of lesions >5	0.452	0.100–2.077	0.268
40–120 Embospheres	0.550	0.219–1.385	0.205
100–300 Embospheres	2.755	0.996–7.585	0.051
300–500 Embospheres	1	NA	NA
500–700 Embospheres	1	NA	NA
100 PVA	1.813	0.394–8.343	0.444
300 PVA	1	NA	NA
Addition of block to embolization	0.427	0.104–1.705	0.226
Addition of biopsy to embolization	1	NA	NA

## Data Availability

Certain data presented in this study are available on request from the corresponding author. The data are not publicly available due to institutional policies.
